# Oral Nutritional Supplements in Adults with Cystic Fibrosis: Effects on Intake, Levels of Fat-Soluble Vitamins, and Bone Remodeling Biomarkers

**DOI:** 10.3390/nu13020669

**Published:** 2021-02-19

**Authors:** Contreras-Bolívar Victoria, Olveira Casilda, Porras Nuria, Abuín-Fernández José, García-Olivares María, Sánchez-Torralvo Francisco José, Girón María Victoria, Ruiz-García Ignacio, Olveira Gabriel

**Affiliations:** 1 Servicio de Endocrinología y Nutrición, Hospital Regional Universitario de Málaga/Universidad de Málaga, Instituto de Investigación Biomédica de Málaga (IBIMA), Málaga 29008, Spain; victoriaconbol@gmail.com (C.-B.V.); nporrasperez@hotmail.com (P.N.); jose.abuin.fdez@gmail.com (A.-F.J.); mery.garcia.96@gmail.com (G.-O.M.); fjstsol@gmail.com (S.-T.F.J.); nach1991@gmail.com (R.-G.I.); 2 Servicio de Neumología, Hospital Regional Universitario de Málaga/Universidad de Málaga, Instituto de Investigación Biomédica de Málaga (IBIMA), Málaga 29008, Spain; marivi_giron@hotmail.com; 3 Centro de Investigación Biomédica en Red–Diabetes y Enfermedades Metabólicas asociadas (CIBERDEM), Barcelona 08036, Spain

**Keywords:** cystic fibrosis, oral nutritional supplements (ONS), nutrition, malnutrition

## Abstract

Background: The use of oral nutritional supplements (ONS) is common practice in patients suffering from cystic fibrosis (CF). We aimed to describe the rate of ONS use to assess their contribution to dietary intake and to determine if they are associated with respiratory status, body composition, muscle strength, bone mineral density (BMD), bone remodeling biomarkers, and plasmatic levels of vitamins. Methods: Cross-sectional study. Patients were clinically stable adults recruited from the CF unit. A 4-day prospective dietary questionnaire was conducted; in addition to respiratory variables, body composition, and BMD (through densitometry, DXA), muscle strength (JAMAR dynamometer), fat-soluble vitamins, and bone remodeling biomarkers (vitamins A, D, and E; osteocalcin, OC; undercarboxylated osteocalcin, ucOC; degradation of the C-terminal telopeptides of type I collagen, CTX; and receptor activator of nuclear factor-kappaB ligand, RANKL) were also evaluated. Results: The study included 59 subjects with CF (57.6% female, mean age 29.3 ± 9.4 years, and BMI 22.0 ± 3.6 kg/m^2^). In this study, 22% (13) patients were taking ONS and presented, compared with those not taking them, significantly more total and mild exacerbations and lower BMI; moreover, they showed a significantly higher total daily calorie intake in addition to a higher consumption of carbohydrates, proteins, and lipids per kg of body weight, omega-3 fatty acids, and vitamins A, D, and E. Vitamin E plasmatic levels were significantly higher in the group on ONS, as was the case with RANKL; finally, a lower rate of vitamin D deficiency was also found. Conclusions: ONS were used by patients with worse respiratory and nutritional statuses and their use was associated with a higher intake of macro- and micronutrients and with better plasmatic levels of fat-soluble vitamins.

## 1. Introduction

Cystic fibrosis (CF) is a disease caused by mutations in a unique gene located on chromosome 7 (the cystic fibrosis transmembrane conductance regulator gene, CFTR) [1].The protein codifying CFTR gene behaves as a chloride channel and mutations cause an epithelial ion transport defect in the respiratory, hepatobiliary, gastrointestinal, and reproductive apparatuses, as well as in the pancreas and sweat glands. 

Until recently, CF was considered to be associated with malnutrition because it was virtually always present at diagnosis and most patients suffered nutritional status impairment, dying severely malnourished. Currently, the prevalence of malnutrition has decreased significantly, although figures are still reported to be around 25% for both adults and children [2]. Malnutrition is a risk factor predictor of morbidity and mortality. The interaction between nutrition and respiratory function is of great relevance, since a parallel decrease in both would negatively affect prognosis and quality of life. Therefore, a nutritional intervention could not only improve nutritional parameters, but also slow down the progressive decline of respiratory function [3,4,5,6,7].

Clinical practice nutrition guidelines recommend a progressive approach to intensify nutritional interventions as the patient’s needs increase, such as preventive nutritional counselling, dietary modifications and/or oral nutritional supplements (ONS), and enteral tube nutrition (preferably through gastrostomy), with a low degree of evidence [3,4,8].

If the nutritional goals are not met or maintained with dietary modifications, administration of pancreatic enzymes, and control of possible comorbidities, then the use of ONS can be indicated; nevertheless, the evidence supporting this approach is low/moderate. ONS can be useful to improve anthropometric parameters (both in adults and children) or several functional issues like spirometry or fatty acid values [9]. Notwithstanding, according to the Cochrane review, ONS do not provide additional benefits regarding the treatment of moderately malnourished children when compared to nutritional counseling and monitoring. Thus, although ONS can be used, they cannot be considered essential and their associated costs have to be taken into account [8].

Our hypothesis is that the use of ONS in selected CF malnourished patients can improve the intake of calories and macro- and micronutrients and reduce vitamin deficiencies.

The objective of our study was to evaluate the rate of ONS used in clinically stable adult patients with CF to assess ONS contribution to dietary intake and to determine if they were associated with changes in respiratory status, body composition, muscle strength, bone mineral density (BMD), levels of fat-soluble vitamins, and bone remodeling biomarkers.

## 2. Materials and Methods

Cross-sectional observational study with a sequential recruitment of patients who attended the CF outpatient consulting room for their annual examination.

### 2.1. Anthropometric and Body Composition Parameters

Weight was assessed through bioimpedance scale mode (TANITA MC980MA, TANITA Corporation, Tokyo, Japan), and height was obtained by a stadiometer (Holtain limited, Crymuch, UK). With these two measurements, body mass index (BMI) was calculated.

Dual-energy X-ray absorptiometry (DXA) was performed using a Lunar Prodigy Advance densitometer (General Electric Medical Systems) to provide information about total body composition (fat-free mass, fat mass, and BMD). The software used was EnCore 12.3 (iDXA and Prodigy Advance). In addition, fat-free mass index (FFMI) was calculated and the prevalence of malnutrition was determined according to the European Society for Clinical nutrition and Metabolism (ESPEN) criteria: <15 (women) or <17 (men) kg/m^2^ [10].

Muscle strength was assessed using an adult dynamometer (Jamar handgrip dynamometry, Asimow Engineering Co., Los Angeles, CA, USA) and data were expressed in absolute figures and compared with the reference population [11].

### 2.2. Dietary Questionnaire

A 4-day prospective dietary questionnaire (including one day of the weekend) was fulfilled by all the participants according to the protocol previously published by our group [12]. The data provided were analyzed by means of a computer application designed by our group for this purpose (Dietstat ^®^, FIMABIS, Málaga, Spain), and the food composition tables of Jiménez and Mataix [13,14] and BEDCA [15] were used. Moreover, the original table of the fat content of fish was substituted by the data of Soriguer et al., which we considered to be more reliable local data [16], and the information on vitamin K (VK) was substituted by the USDA database [17]. The composition of any nutritional supplements that patients were taking was also included in the database. The supplements were chosen based on the clinical characteristics of the patients and taking into account their personal preferences.

Furthermore, data corresponding to micronutrient supplementation (fat-soluble vitamins and minerals) were also collected.

### 2.3. Laboratory Measurements

A complete blood test was performed to assess coagulation, biochemical values, HbA1c, fat-soluble vitamins (A, D, and E), bone remodeling biomarkers (degradation of the C-terminal telopeptides of type I collagen, CTX; receptor activator of nuclear factor-kappaB ligand, RANKL; osteocalcin, OC; and undercarboxylated osteocalcin, ucOC).

Blood samples were collected after a 12-h fast on the same day as the bone densitometry. The extractions were completed after a clinical examination to confirm that the patients were in a stable phase. Plasma and serum were separated into aliquots and stored until analysis at −80 °C in the Hospital-IBIMA biobank, which belongs to the biobank of the public health system of Andalusia (BSSPA) and to the Spanish National Biobank Platform (PT13/0010/0006). Fat-soluble vitamins A and E were determined through HPLC (High Performance Liquid Chromatography, Agilent 1200 of Bio-Rad, Hercules, CA, USA). Vitamin D was analyzed by electrochemiluminescent immunoassay (Modular E-170, Roche Diagnostics). The serum levels of bone remodeling biomarkers were determined by enzyme immunoassay techniques: ucOC (TAKARA Bio INC, Shiga, Japan) (intra-assay CV = 5.1%; inter-assay CV = 8.3%), OC (R&D System, Minneapolis, USA.) (intra-assay CV = 3,1%; inter-assay CV = 7.2%), CTX (Serum CrossLaps, IDS-UK) (intra-assay CV = 7.1%; inter-assay CV = 6.2%), and RANKL (InmmunoDiagnostics System, Frankfurt, Germany) (intra-assay CV = 6.4%; inter-assay CV = 5.6%). All these determinations were quantified in Versamax (MTX Lab System, Barcelona, Spain).

### 2.4. Assessment of Respiratory Status

The exacerbations recorded during the annual examination were assessed taking into consideration those that took place in the year prior to the evaluation. Such exacerbations were classified into mild/moderate (suggestive symptoms and blood test results, not requiring hospitalization, and resolved with oral antibiotics) or severe (suggestive symptoms that worsened and required hospitalization and/or intravenous antibiotics on an outpatient basis) [18]. Moreover, patients underwent forced spirometry via a JAEGER pneumotachograph (Jaeger Oxycon Pro^®®^ Erich Jaeger, Würzberg, Germany) following the SEPAR guidelines, and values of forced vital capacity (FVC), maximum expiratory volume in the first second (FEV1), and the ratio between both (FEV1/FVC) were determined. The values were expressed in absolute terms in mL and as a percentage of the theoretical value for the same age, weight, and height of subjects according to a reference population [19]. Bronchorrhea, the amount of sputum produced per day expressed in milliliters, was evaluated according to the estimate of the patient during the three days prior to the visit [18].

### 2.5. Statistical Analyses

Data were processed with SPSS 22.0 (SPSS Inc., Chicago, IL, USA, 2013). Quantitative variables were expressed as the mean ± standard deviation and as 95% confidence intervals. Comparison between qualitative variables was done with the Chi-square test with Fisher’s test when necessary.

The distribution of quantitative variables was examined using the Kolmogorov–Smirnov test. Comparison of quantitative variables between the two groups was done with the Student t-test, and the Mann–Whitney nonparametric test was used for variables not following a normal distribution.

For all the calculations, the significance was set at *p* < 0.05 for two-tailed tests.

### 2.6. Ethics

All subjects gave their informed consent for inclusion before they participated in the study. The study was conducted in accordance with the Declaration of Helsinki, and the protocol was approved by the Research Ethics Committee of Malaga Province (27 April 2017).

## 3. Results

A total of 59 CF patients were recruited: 25 (42.4%) males and 34 (57.6%) females. Their mean age was 29.3 ± 9.4 years (range 16–67) and their general characteristics are displayed in Table 1.

Only 13 (22%) patients were taking ONS: 3 patients were taking Diben drink^®^, 3 patients Fresubin energy drink^®^, 1 patient Resource HPHC^®^, 1 patient Diasip^®^, 1 patient Fortisip^®^, 1 patient Resource CF^®^, 1 patient Duocal^®^, 1 patient Resource diabet^®^ and 1 patient Glucerna SR^®^.

They presented more severe respiratory symptoms and worse nutritional status compared to the other patients. They presented significantly more total and mild respiratory exacerbations with a non-significant trend towards lower spirometry and more daily bronchorrhea. Furthermore, their BMI was significantly lower (20.08 ± 2.30 vs. 22.51 ± 3.72, *p* < 0.03) and their FFMI tended to present lower values. These patients also had a non-significant trend toward lower BMD. Moreover, these patients had a higher calorie intake (daily and per kg of body weight) than patients not taking ONS. They also consumed more carbohydrates, proteins, and lipids per kg of body weight, omega-3 fatty acids, and vitamins A, D, and E. When we analyzed the total vitamin consumption (food + ONS + micronutrient supplements), we found that patients on ONS took a higher amount of vitamins A, D, and K. The only difference regarding bone remodeling biomarkers was that patients on ONS had significantly higher levels of RANKL. Finally, a lower rate of vitamin D deficiency and better plasmatic levels of vitamin E were also found and no patient taking ONS had a deficiency of vitamin D or E. (Table 1, Table 2, Table 3 and Table 4).

In Table 5, the macro- and micronutrients obtained from ONS are shown:

## 4. Discussion

In this study, 22% of the patients were taking ONS, and they presented more severe respiratory symptoms and worse nutritional status compared to the other patients. ONS consumption was associated with higher energy intake and macro- and micronutrients consumption, as well as with an increase in plasmatic levels of fat-soluble vitamins E and D.

There is a current lack of randomized, prospective, controlled studies evaluating the efficacy of nutritional interventions using supplements in patients with CF [8]. Moreover, for ethical reasons, no studies in which the control group does not receive any nutritional intervention (patients needing it) have been performed and the use of behavior therapy (changes in dietary habits) has not been sufficiently compared with the prescription of ONS. In this context, the current guidelines from Europe, United Kingdom, and USA provide recommendations based on expert consensus [3,4]. In short, these guidelines recommend a progressive approach to intensify nutritional interventions as the patient’s needs increase: preventive nutritional counselling, dietary modifications and/or oral nutritional supplements (ONS), and enteral tube nutrition (preferably through gastrostomy) with a low degree of evidence [3,4,8].

There are clinical situations in which ONS could be helpful to increase daily energy intake and to improve malnutrition, for example, in patients on transplant waiting lists, or in cases of severe respiratory exacerbations or chronic deterioration of nutritional status not improving with dietary recommendations or behavior therapy [20]. It is important to make an accurate assessment of the indication by fostering personalized dietary recommendations and excluding other possible causes that may be contributing to malabsorption or to reduced calorie intake (such as digestive complications, incorrect use of pancreatic enzymes, eating disorders, uncontrolled diabetes, etc.) [21].

The short-term use of ONS can increase total energy intake without significantly reducing the calorie intake from food [22]. ONS can be also useful to improve anthropometric parameters (both in adults and children) or several functional issues like spirometry, fatty acid values, or physical activity level [9]. Notwithstanding, ONS may not provide additional benefits regarding the treatment of moderately malnourished subjects compared to nutritional counselling and medium- and long-term monitoring. Thus, it is convenient to periodically assess their indication [4,8]. Moreover, to assure that ONS are used to complement (and not to replace) meals, as evidenced in the CALICO study (Calories in Cystic Fibrosis-Oral) [23,24], it is relevant to pay attention to the quantity and moment of consumption of these supplements. In this sense, the wide variety of presentations and flavors can help to minimize the fatigue that is often associated with long-term use of ONS. Furthermore, treatment compliance has to be periodically assessed as a certain percentage of patients end up discontinuing treatment [25].

In our study, patients taking ONS showed a worse respiratory status as they had more exacerbations and trended towards lower FEV1 (%). Furthermore, their BMI was significantly lower and their FFMI tended toward lower values. In our series, the proportion of FC patients taking ONS was lower than the rate previously described based on data from more than 300 patients from 10 Spanish centers (44% vs. 22%), although no direct comparison can be done due to our small sample size [26].

Malnourished patients have a higher risk of osteopenia and osteoporosis, and it would be expected to find increased bone remodeling biomarkers. In our study, patients taking ONS tended to present a lower BMD, although this was not significant (possibly due to the small sample size), and significantly higher levels of RANKL. When RANKL is overexpressed, it favors the development of osteopenia and osteoporosis in CF patients, promoting the differentiation and activation of osteoclasts [27]. On the contrary, we did not find differences regarding other remodeling markers, including levels of ucOC, which increase in the case of vitamin K (VK) deficiency [28]. VK behaves as a cofactor for some proteins that participate in bone mineralization, like OC, and in bone matrix calcification regulation. Similarly, VK inhibits the production of prostaglandin E2 and interleukin 6, which are bone-resorptive agents (246); VK supplementation can help to improve BMD and reduce fracture risk [29,30]. In this sense, the use of ONS could improve nutritional status and reduce the loss of bone mass in malnourished CF patients through several mechanisms, for example, by providing fat-soluble vitamins. The prevalence of fat-soluble deficiency/insufficiency in FC patients is estimated to range between 10% and 40% for vitamin A, between 22% and 90% for vitamin D (establishing as normal values higher than 30 ng/mL), around 23% in recently diagnosed children, and around 14% in adolescents/adults for vitamin E; for VK, it could reach up to 60–70% using markers like proteins induced by vitamin K absence-II (PIVKA-II) and ucOC [4,31,32]. Therefore, despite the worse respiratory and nutritional status, patients taking ONS presented a higher intake of calories and macronutrients as well as higher levels of fat-soluble vitamins D and E. No patient taking ONS had deficiency of vitamin D or E. These results suggest that ONS could not only be substituting patients’ dietary intake, but also supplementing their normal diet.

The inclusion of the new CFTR protein repair therapy (potentiators and correctors) has significantly and clinically improved lung function, and it may also improve bone status (Z-score), insulin secretion, and nutritional status (through several mechanisms such as fat absorption, reduction of energy consumption and gut inflammation, or microbiota modifications). Its use is likely to (in the near future) modify the natural history of the disease and to have an effect on the necessity of ONS prescription and its efficacy in the prevention or treatment of malnutrition in CF patients.

The strengths of our study are related to the integrated use of multiple parameters to assess nutritional status: 4-day dietary questionnaire, body composition, bone status (using DXA), laboratory data (levels of vitamins, bone remodeling markers), and respiratory function. Nevertheless, there are some limitations. It is a cross-sectional study, which hampers the drawing of causal conclusions and is why we can only discuss associations. It is also a single center study that includes a small number of participants, which impedes generalizing the results obtained.

## 5. Conclusions

In conclusion, the proportion of CF patients taking ONS is lower than the rate previously described in other series and was taken by the most severe patients (worse respiratory and nutritional status). ONS use was associated with a higher intake of macro- and micronutrients and with better levels of fat-soluble vitamins. This study may lay the groundwork for future clinical trials and evaluating the role of ONS in people suffering from CF.

## Figures and Tables

**Table 1:** General characteristics and respiratory status.

		CF	CF		
		Total	No ONS (*n =* 46)	ONS (*n* = 13)	*p*
Age	(m ± SD)	29.3 ± 9.4	29.8 ± 9.7	27.4 ± 8.2	NS
Sex					NS
Male Female	*n* (%)	25 (42.4) 34 (57.6)	17 (36.9) 29 (63)	8 (61.5) 5 (38.4)	
Respiratory status					
Bronchorrhea (mL)	(m ± SD)	21.3 ± 20.8	19.7 ± 19.2	25.8 ± 23.2	NS
Total exacerbations	(m ± SD)	2.3 ± 1.8	2.0 ± 1.5	3.6 ± 2.2	<0.01
Mild exacerbations	(m ± SD)	1.9 ± 1.6	1.6 ± 1.3	3.2 ± 1.8	<0.01
Severe exacerbations	(m ± SD)	0.41 ± 0.80	0.4 ± 0.8	0.5 ± 0.8	NS
FEV1 (%)	(m ± SD)	63.3 ± 25.6	66.9 ± 25.1	51.6 ± 24.9	NS
FVC (%)	(m ± SD)	75.3 ± 22.0	77.5 ± 22.3	66.9 ± 19.6	NS
FEV1/FVC (%)	(m ± SD)	66.7 ± 12.0	68.0 ± 15.7	61.4 ± 17.8	NS
Colonisations					
Colonisation by *S. Aureus*	*n* (%)	47 (79.7)	35 (76.1)	12 (92.3)	NS
Colonisation by *H. influenzae*	*n* (%)	28 (47.5)	19 (41.3)	9 (69.2)	NS
Colonisation by *P. aeruginosa*	*n* (%)	47 (79.7)	36 (78.3)	11 (84.6)	NS

Maximum expiratory volume in the first second (FEV1); Forced vital capacity (FVC), m ± SD: mean ± standard deviation; NS: Not significant; p: statistical significance between oral nutritional supplements (ONS) and No ONS.

**Table 2:** Bone and nutritional status.

		CF	CF		
		Total	No ONS (*n* = 46)	ONS (*n* = 13)	*p*
Bone status					
BMD (g/cm^2^)	(m ± SD)	1.090 ± 0.2330	1.092 ± 0.250	1.086 ± 0.175	NS
T-score	(m ± SD)	−0.532 ± 1.295	−0.48 ± 1.25	−0.76 ± 1.55	NS
Z-score	(m ± SD)	−0.502 ± 1.185	−0.46 ± 1.09	−0.64 ± 1.49	NS
Normal	*n* (%)	39 (66.1)	32 (69.5)	7 (53.8)	NS
Osteopenia	*n* (%)	15 (25.4)	11 (23.9)	4 (30.8)	
Osteoporosis	*n* (%)	5 (8.5)	3 (6.6)	2 (15.4)	
Nutritional status			*n* = 40	*n* = 12	
BMI	(m ± SD)	22.0 ± 3.6	22.5 ±3.7	20.1 ±2.3	<0.05
Fat mass (kg)	(m ± SD)	15.9 ± 8.5	16.8 ± 8.9	12.4 ± 6.1	NS
Fat-free mass (kg)	(m ± SD)	42.8 ± 9.4	43.2 ± 9.9	41.5 ±7.8	NS
Fat mass (%)	(m ± SD)	26.3 ± 10.7	27.3 ± 10.4	23.0 ± 11.6	NS
Fat-free mass (%)	(m ± SD)	73.7 ± 10.7	72.7 ±10.4	76.9 ±11.6	NS
FFMI (kg/m^2^)	(m ± SD)	16.3 ± 2.4	16.5 ± 2.4	15.6 ± 2.3	NS
Malnourished (FFMI <17 M/15F)	*n* (%)	29	20 (50)	9 (75.3)	NS
Strength			*n* 46	*n* 13	
Hand-grip dynamometry	(m ± SD)	30.3 ± 7.3	30.4 ± 11.9	29.5 ± 9.5	NS

BMD: bone mineral density; FFMI: fat-free mass index; FFMI <17 M/15 F: fat-free mass index <17 kg/m^2^ in males and <15 kg/m^2^ in females. m ± SD: mean ± standard deviation; NS: Not significant. p: statistical significance between ONS and No ONS.

**Table 3:** Dietary questionnaire and fat-soluble vitamin supplementation. Association between dietary questionnaire and ONS intake.

		CF	CF		
		Total	No ONS *(n* = 46)	ONS (*n* = 13)	*p*
DIETARY QUESTIONNAIRE (*n* = 59) *					
Mean calorie intake	(m ± SD)	2663.4 ± 574.3	2456.0 ± 497.3	2982.4 ± 553.3	<0.01
Calories/kg of body weight	(m ± SD)	47.4 ± 11.7	41.3 ± 9.0	56.8 ± 8.9	<0.001
Calories from supplements (% of total)	(m ± SD)		-	16.0 ± 10.4	-
Carbohydrates (%)	(m ± SD)	44.7 ± 5.3	45.1 ± 6.0	44.0 ± 4.3	NS
Carbohydrates/kg of body weight	(m ± SD)	5.3 ± 1.4	4.7 ± 1.2	6.2 ± 1.1	<0.01
Proteins (%)	(m ± SD)	16.1 ± 2.4	15.2 ± 2.2	17.3 ± 2.2	<0.05
Proteins/kg of body weight	(m ± SD)	1.9 ± 0.6	1.6 ± 0.4	2.4 ± 0.4	<0.001
Lipids (%)	(m ± SD)	38.0 ± 8.4	39.4 ± 5.3	35.9 ± 11.6	NS
Lipids/kg of body weight	(m ± SD)	2.1 ± 0.6	1.8 ± 0.4	2.4 ± 0.5	<0.001
Omega 3 fatty acids	(m ± SD)	1.5 ± 1.1	1.08 ± 0.45	2.27 ± 1.38	<0.01
Omega 6/Omega 3	(m ± SD)	8.2 ± 4.2	10.1 ± 4.1	5.4 ± 2.3	<0.001
Vitamin A (UI)	(m ± SD)	1364.5 ± 778.0	936.2 ± 526.7	2023.4 ± 631.7	<0.001
Vitamin D (UI)	(m ± SD)	1187.1 ± 1670.5	532.5 ± 776.1	2194.1 ± 2162.2	<0.05
Vitamin E (mg)	(m ± SD)	17.3 ± 8.1	13.4 ± 5.1	23.4 ± 8.2	<0.01
Vitamin K (mcg)	(m ± SD)	138.8 ± 123.4	121.6 ± 130.5	182.5 ± 93.8	NS
TOTAL FAT-SOLUBLE VITAMINS **					
Vitamin A (UI)	(m ± SD)	6403.9 ± 4379.8	5056.2 ± 4188.7	8477.2 ± 3961.1	<0.05
Vitamin D (UI)	(m ± SD)	3471.0 ±2797.8	2370.7 ± 2169.4	5163.9 ± 2880.8	<0.01
Vitamin E (mg)	(m ± SD)	267.3 ± 169.6	241.4 ± 179.1	307.2 ± 152.0	
Vitamin K (mcg)	(m ± SD)	1045.7 ± 1556.5	718.3 ± 716.7	1851.5 ± 2562.5	<0.05

* (Including food + ONS); ** Including food + ONS + Micronutrient supplements. m ± SD: mean ± standard deviation; NS: Not significant; p: statistical significance between ONS and No ONS.

**Table 4:** Laboratory data. Association between blood test values and ONS intake.

		CF	CF		
		Total	No ONS (*n* = 46)	ONS (*n* = 13)	*p*
Blood test data (*n* = 59)					
Vitamin A (mcg/dl)	(m ± SD)	46.4 ± 20.1	46.4 ± 19.8	46.6 ± 21.9	NS
<20	n (%)	2 (3.4)	1 (2.2)	1 (7.7)	NS
25-OH-Vitamin D (mcg/dl)	(m ± SD)	38.1 ± 28.6	37.4 ± 31.3	40.6 ± 15.1	NS
<20	n (%)	13 (22.0)	13 (28.3)	0 (0)	<0.05
>30	n (%)	29 (49.2)	21 (45.6)	8 (61.5)	
Vitamin E (mg/g)	(m ± SD)	1248.2 ± 413.8	1174.3 ± 396.6	1488.3 ± 390.9	<0.05
<800	n (%)	6 (10.2)	6 (12.8)	0 (0)	NS
Vitamin E/Cholesterol	(m ± SD)	9.18 ± 3.30	8.3 ± 2.9	12.0 ± 3.0	<0.001
<5,7	n (%)	2 (3.4)	2 (5.3)	0 (0)	NS
Prothrombin time (%)	(m ± SD)	97.1 ± 11.9	97.4 ± 11.8	95.7 ± 13.3	NS
ucOC (ng/mL)	(m ± SD)	4.7 ± 2.7	4.7 ± 2.7	4.7 ± 2.4	NS
OC (ng/mL)	(m ± SD)	26.4 ± 25.9	27.5 ± 26.7	22.0 ± 23.4	NS
CTX (mcg/mL)	(m ± SD)	0.55 ± 0.30	0.53 ± 0.30	0.63 ± 0.35	NS
RANKL (pmol/L)	(m ± SD)	0.23 ± 0.28	0.18 ± 0.22	0.42 ± 0.42	<0.05

CTX: degradation of the C-terminal telopeptides of type I collagen; RANKL: receptor activator of nuclear factor-kappaB ligand; OC osteocalcin; ucOC: undercarboxylated osteocalcin; m ± SD: mean ± standard deviation; NS: Not significant; p: statistical significance between ONS and No ONS.

**Table 5:** Macro- and micronutrients obtained from ONS.

		CF
		ONS (13)
DIETARY QUESTIONNAIRE		
Mean ONS per day	(m ± SD)	1.8 ± 1.1
Mean calorie intake	(m ± SD)	479.5 ± 326,1
Calories from supplements (% of total)	(m ± SD)	16.0 ± 10.4
Carbohydrates (%)	(m ± SD)	43.2 ± 6.6
Carbohydrates (g)	(m ± SD)	56.0 ± 39.9
Proteins (%)	(m ± SD)	16.9 ± 3.1
Proteins (g)	(m ± SD)	21.6 ± 13.9
Lipids (%)	(m ± SD)	40.0 ± 5.4
Lipids (g)	(m ± SD)	22.6 ± 14.4
Omega 3 fatty acids	(m ± SD)	1.1 ± 1.2
Omega 6/Omega 3	(m ± SD)	0.3 ± 0.2
Vitamin A (UI)	(m ± SD)	560.2 ± 59.4
Vitamin D (UI)	(m ± SD)	219.6 ± 151.6
Vitamin E (mg)	(m ± SD)	10.5 ± 7.1
Vitamin K (mcg)	(m ± SD)	41.5 ± 31.6

## Data Availability

The data presented in this study are available on request from the corresponding author.
